# Editorial Topical Collection: “Explainable and Augmented Machine Learning for Biosignals and Biomedical Images”

**DOI:** 10.3390/s23249722

**Published:** 2023-12-09

**Authors:** Cosimo Ieracitano, Mufti Mahmud, Maryam Doborjeh, Aimé Lay-Ekuakille

**Affiliations:** 1 DICEAM Department, University Mediterranea of Reggio Calabria, Via Zehender, Feo di Vito, 89122 Reggio Calabria, Italy; 2 Department of Computer Science, Nottingham Trent University, Nottingham NG11 8NS, UK; mufti.mahmud@ntu.ac.uk; 3 Computing and Informatics Research Centre, Nottingham Trent University, Nottingham NG11 8NS, UK; 4 Medical Technologies Innovation Facility, Nottingham Trent University, Nottingham NG11 8NS, UK; 5 Computer Science and Software Engineering, Auckland University of Technology, Auckland 1010, New Zealand; maryam.gholami.doborjeh@aut.ac.nz; 6 Department of Innovation Engineering, University of Salento, 73100 Lecce, Italy; aime.lay.ekuakille@unisalento.it

Machine learning (ML) is a well-known subfield of artificial intelligence (AI) that aims at developing algorithms and statistical models able to empower computer systems to automatically adapt to a specific task through experience or learning from data [1]. ML techniques have been demonstrating remarkable breakthroughs in the field of biomedical research, especially in predictive analytics and classification tasks [2,3].

However, the success of ML in this domain has been accompanied by a challenge, i.e., the inherent opaqueness of ML algorithms [4]. Indeed, despite their efficacy, ML algorithms lack transparency in their decision-making processes and are often seen as black boxes. This lack of transparency raises concerns, especially in critical domains, where understanding the rationale behind machine decisions is important for fostering trust in decision-making [5].

In this regard, the emergence of explainable artificial intelligence (xAI) techniques has become a pivotal focus within this field. In particular, xAI methods strive to unveil the internal mechanisms of the AI algorithms, aiming to shed light on the outcomes, predictions, decisions, and recommendations generated by such models [6,7]. The primary objective is to enhance the interpretability and transparency of machine decisions. This is of paramount importance in medical applications, where such enhanced comprehension could have a significant impact on clinicians’ final decision-making [8]. In this context, several xAI-based approaches have been emerging in clinical applications, for example, rehabilitation systems based on brain–computer interfaces [9], detection of neurological disorders [10], breast cancers [11,12], and medical imaging analysis [13].

Furthermore, the escalating availability of medical and clinical data, collected from an expanding network of interconnected biosensors within the Internet of Things (IoT) framework, provides a rich source for training and refining ML models [14,15]. In addition, recent advances in augmented techniques, i.e., generative adversarial networks (GANs), have enhanced the decision-making capabilities of ML algorithms. Indeed, generative models are able to produce synthetic samples, augmenting the training data, potentially addressing issues related to data scarcity, and improving the generalization of models [16,17].

In this context, this topical collection includes ten papers focused on the latest advancements in the field of explainable and augmented ML applied to biosignals and biomedical images. Each of the ten original contributions accepted for publication has undergone a rigorous review process by a minimum of two expert reviewers across at least two rounds of revision. These studies published in the current topical collection are briefly summarized as follows:

In contribution 1, the authors developed a brain-inspired neural network to explore the effect of mindfulness training on the electroencephalographic (EEG) function. In particular, a spiking neural network (SNN) was employed to assess the neural patterns generated over both spatial and temporal features derived from EEG data, which captured the neural dynamics linked to event-related potentials (ERPs). Furthermore, the interpretability of the SNN model was also further investigated. Outcomes indicated that SNN models provide valuable insights in distinguishing between different brain states in response to specific tasks and stimuli, as well as tracking changes in brain states through psychological interventions.

In contribution 2, a novel explainable analysis of potential biomarkers denoting tumorigenesis in non-small cell lung cancer is proposed based on detailed mathematical formulation for mRNA, ncRNA, and mRNA–ncRNA regulators. Specifically, the authors developed a system involving coupled-reaction partial differential equations to model temporal gene expression profiles within a two-dimensional spatial domain, capturing the transition states before converging to the stationary state. Experimental results demonstrate that the mathematical gene-expression profile provides the most accurate fit for the population abundance of these oncogenes.

In contribution 3, Vargas-Lopez et al. introduced an explainable machine learning approach that employed statistical indexes and support vector machines (SVMs) to detect stress in automobile drivers based on electromyographic (EMG) signals. The authors investigated the efficacy of seventeen statistical time features and, based on the analysis of the results, concluded that combining variance and standard deviation with a support vector machine classifier utilizing a cubic kernel is an effective approach for detecting stress events, achieving an AUC of 0.9.

In contribution 4, the authors conducted an extensive analysis of the most effective methods for classifying the emotion of fear, encompassing a range of machine learning methods such as decision trees, k-Nearest Neighbors, support vector machines, and artificial networks. In addition, xAI was also explored by means of Local Interpretable Model-Agnostic Explanations in order to interpret and justify predictions in a human-understandable manner. Experimental results showed classification performance, achieving accuracy from 91.7% using to 93.5% using dimensionality reduction and SVM.

In contribution 5, Doborjeh et al. introduced an innovative methodology aimed at enhancing the interpretability of a brain-inspired SNN for deep learning and knowledge extraction. Their methodology focused on the learning process from real-time spatiotemporal brain data in an incremental and online operational mode. The experimental results show that by selecting a specific group of EEG features, the accuracy of EEG classification could be enhanced to 92%, outperforming all-feature-based classification.

In contribution 6, a novel approach for assessing the degree of gait impairment in Parkinson’s disease using a computer vision-based approach was proposed. In addition, the interpretability of the feature values could be used by clinicians to support their decision-making and provide insight into the model’s objective UPDRS rating estimation.

In contribution 7, the authors explored several xAI techniques such as GradCAM, LIME, RISE, Squaregrid, and direct gradient approaches with the ultimate aim of further explaining COVID-19 CT-Scan classifiers. Experimental results reported that VGG16 was the most affected by biases related to misleading artifacts, whereas DenseNet was more robust against them. In addition, it was observed that even slight differences in validation accuracies could lead to significant alterations in the explanation heatmaps for DenseNet architectures.

In contribution 8, Usama et al. developed AI-based classifiers for classifying single-trial error-related potentials (ErrPs) produced by twenty-five subjects with stroke. Specifically, EEG recordings were partitioned into epochs (ErrPs and NonErrPs) and classified by means of multi-layer perceptron based on temporal features or the entire epoch. Moreover, feature classification was also conducted using shrinkage LDA. The authors concluded that by employing physiological brain potentials (ErrP and NonErrP) as input to the classifiers, it may be possible to interpret the classifier outputs in the context of established physiological research within this domain.

In contribution 9, Seven et al. proposed a novel pipeline for xAI imaging based on radiomic features and Shapley values for explaining predictions achieved by complex models. In particular, the authors conducted a retrospective analysis of data from glioma patients and presented an explainable prediction model for identifying isocitrate dehydrogenase mutations using radiomics data. Such a model could serve as a valuable tool in clinical decision-making.

In contribution 10, the authors developed an interpretable diabetes detection system using an xAI. To this end, the Pima Indian diabetes dataset was employed, and six ML algorithms were implemented along with an ensemble classifier to diagnose the diabetes disease. Global and local explanations were performed by means of the Shapley additive explanations (SHAP). The results reported accuracy of 90% and an F1 score of 89% using a five-fold cross-validation.

In summary, this topical collection has tackled numerous significant challenges in xAI and has presented innovative computational methods with potential deployment in clinical contexts. We would like to express our deepest gratitude to *Sensors* journal’s Managing Team for their continuous support throughout the preparation of this collection. We greatly thank all the contributing authors and the anonymous expert reviewers whose invaluable efforts helped to select the submissions with the utmost quality.
